# Features of interest from a multi-season satellite survey of baleen whales on the West Antarctic Peninsula

**DOI:** 10.1038/s41597-025-06463-x

**Published:** 2025-12-31

**Authors:** C. C. G. Bamford, H. Cubaynes, N. Kelly, P. J. Clarke, E. Longden, M. Weyn, G. Perry, H. Snead, L. Fouda, G. Macfarlane, J. A. Jackson

**Affiliations:** 1 British Antarctic Survey, High Cross, Madingley Road, CB3 0ET Cambridge, UK; 2 Australian Antarctic Division, Department of Agriculture, Water and the Environment, Australian Government, Channel Highway, Kingston, 7050 Australia; 3 School of Engineering, University of Edinburgh, Sanderson Building, Robert Stevenson Road, The King’s Building, Edinburgh, EH9 3FB UK; 4 Sea Mammal Research Unit, Scottish Oceans Institute, University of St Andrews, St Andrews, Fife KY16 9XL UK; 5 MARE – Marine and Environmental Sciences Centre/ARNET – Aquatic Research Network, Regional Agency for the Development of Research, Technology and Innovation (ARDITI), Funchal, Madeira Portugal; 6 MARE – Marine and Environmental Sciences Centre/ARNET – Aquatic Research Network, Institute for Research and Advanced Training (IIFA), University of Évora, Évora, Portugal; 7 Faculty of Life Sciences, University of Madeira, Funchal, Madeira Portugal; 8 Department of Biology, University of Évora, Pólo de Mitra, Apartado 94, Évora, 7002-554 Portugal; 9 Royal Society for the Protection of Birds, The Lodge, Potton Road, Sandy, SG19 2DL UK; 10 APEM Ltd, Riverview, A17 Embankment Business Park, Heaton Mersey, Stockport, SK4 3GN UK; 11 Department of Biological Sciences, University of New Brunswick, Saint John, NB Canada; 12 Department of Geography, Simon Fraser University, Burnaby, BC V5A 1S6 Canada

**Keywords:** Marine biology, Conservation biology

## Abstract

The application of very high-resolution satellite imagery for the purpose of studying wildlife, particularly in remote regions, has gained significant traction in recent years. With this, there has been an exponential increase in the volume of satellite data collected, which has fostered a shift towards the use of automated systems to increase processing efficiency. However, these automated systems require manually annotated data on which to be trained, which is lacking due to the time required to manually annotate satellite imagery and the lack of published records to collaboratively build large enough training datasets. Here, we present a dataset that describes a total of 819 annotated and classified Features of Interest (FOIs) from a multi-season baleen whale-focussed survey of Wilhelmina Bay on the Western Antarctic Peninsula. These data are comprised of FOIs that have been annotated and classified based on existing protocols by seven individual observers who scanned ~1,900 km^2^ of WorldView-3 imagery acquired between 2018 and 2022 to expedite the creation of training datasets for automated detection models.

## Background & Summary

Since its first implementation^1,2^ the usage of very high-resolution (VHR) satellite imagery as a means to study remote wildlife populations has grown in popularity over recent years. To date, this format of data collection has been applied to a wide range of species, from whales^2–5^ and seals^6–8^ to African herbivores^9,10^, birds^11,12^ and species at both poles^13–19^. Incremental improvements in the spatial resolution of the imagery have aided with detection certainty, whereby features in higher resolution images are now clearer to detect and features, such as baleen whales and their features (i.e., flippers, flukes, blows etc.), can now be more reliably identified, particularly in complex marine environments^20^.

A factor that commonly limits the inclusion of satellite imagery in ecological studies is the initial manual effort required to examine large areas of imagery^2–5^, and the upfront cost associated with licensing imagery from satellite companies. Automated approaches are increasingly being implemented to reduce this workload^21,22^. However, the success of these automated approaches is often limited by the availability of training data, which these algorithms use to repeatedly test search parameters against^23^. The availability of such training data, particularly for baleen whales in the polar regions, is limited^24^ due to the manual effort required to process the imagery, along with complications associated with sharing raw imagery, which is under licence from the satellite company and cannot be disseminated in its native format (i.e., GeoTIFF). The lack of availability of training data is especially pertinent for whales, as the visibility of these animals in satellite imagery is influenced by the characteristics of their environment, and training data available from one region may not be appropriate at informing the search parameters for another, particularly when water colour and turbidity influence detection^20,25^. Here, we present an annotated dataset containing features of interest (FOIs) located by seven observers viewing WorldView-3 imagery of a coastal embayment of the Western Antarctic Peninsula (WAP) between 2018 and 2022.

## Methods

### Study area

**Fig. 1:**
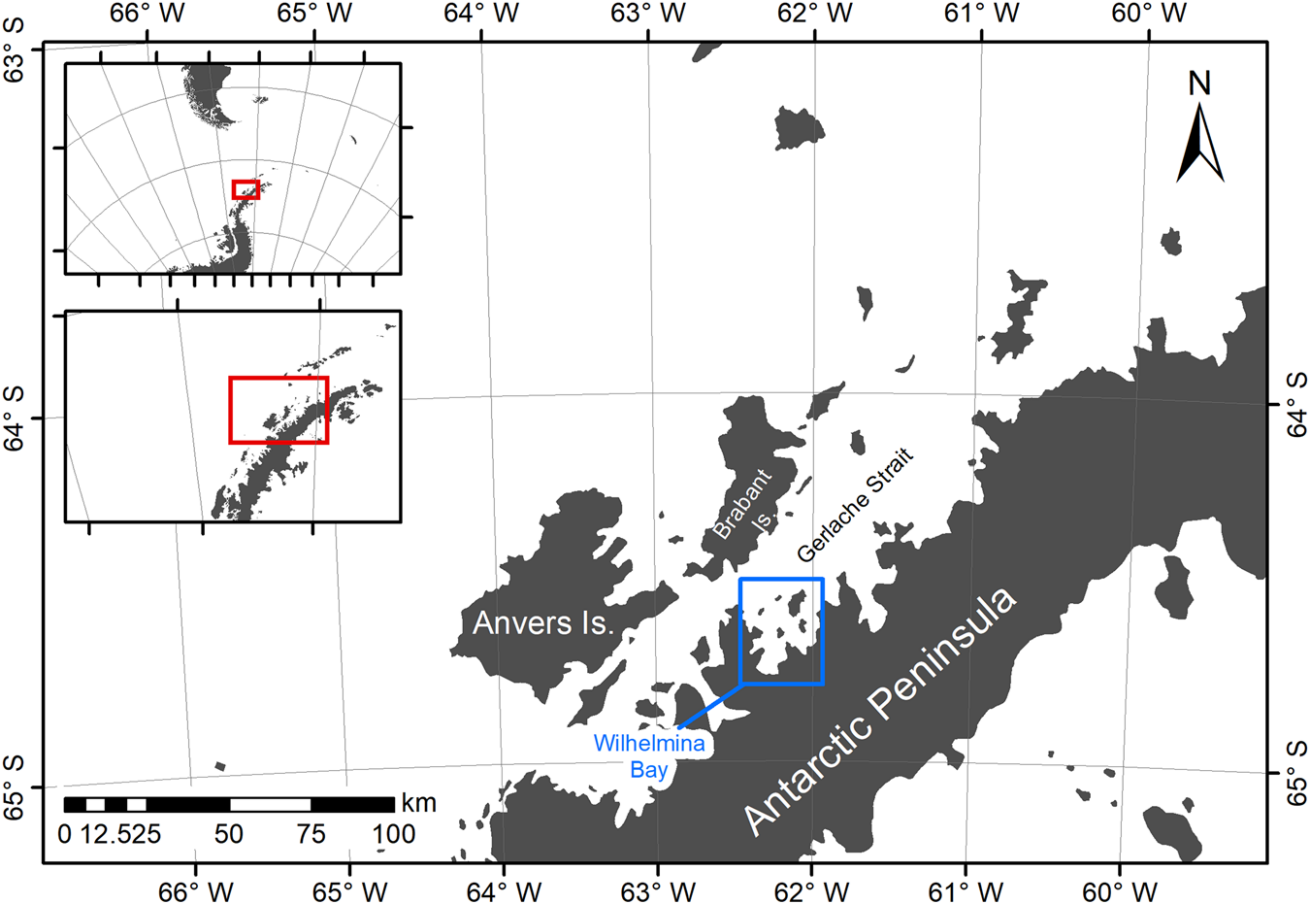
Acquisition region of the tasking of very-high-resolution WorldView-3 satellite imagery over Wilhelmina Bay (blue) on the Western Antarctic Peninsula.

**Fig. 2:**
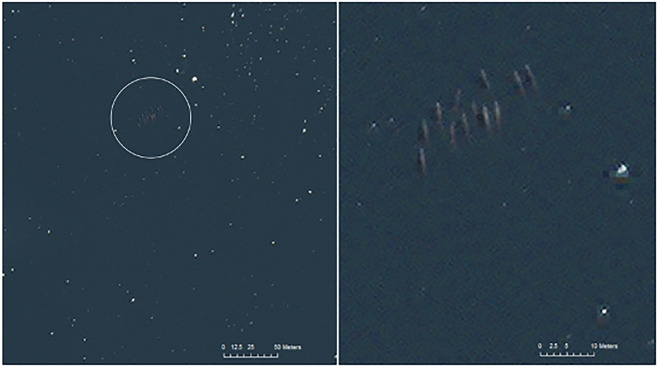
A pod of 12 cetaceans present in the image taken on the 21.02.2019, likely beaked whales – Southern bottlenose (*Hyperoodon planifrons*) or Arnoux’s beaked whales (*Berardius arnuxii*). Contextual panel on the left and zoomed panel on the right. Satellite imagery © Maxar Technologies.

The complex fjord-like network of channels and bays of the WAP, all have the potential to support whales. In selecting our study site, we considered several factors: (i) existing knowledge of the density of whales in particular regions^26,27^; (ii) how long the region would remain ice free and have suitable light conditions to maximise the length of the study; and (iii) assessed how likely it was for suitable conditions for detecting whales to occur^2,4^. Based on these, we selected Wilhelmina Bay (64°37′S 62°10′W, Fig. 1). This embayment opens from the Gerlache Strait and is known for its high abundance of whales^26,27^. Accurate species identification of baleen whales in satellite imagery, in a mixed-species environment, is challenging, and as a result we chose a region where one species predominates, which in these waters are humpback whales (*Megaptera novaeangliae*)^4,26–28^. Other identified whales, in this case, smaller cetaceans, were easily differentiated based on behavioural or physiological characteristics visible in the imagery^3,4,29^ and, when identified, were removed from inclusion in the presented data (Fig. 2).

### Image acquisition

WorldView-3 images (Standard 2 A product type and level) were tasked and purchased with a panchromatic resolution of 0.31 m and a multispectral resolution of 1.24 m from Maxar Technologies. We acquired images over three seasons: (i) 2018 to 2019; (ii) 2019 to 2020; and (iii) 2021 to 2022, from the earliest weather window through until acquisitions were not possible due to light levels. This gave us a range of 114, 108 and 122 days from first to last image acquisition for each of the three seasons, respectively (Table 1).

**Table 1:** Dates and catalogue IDs of VHR images areas collected over the Austral summer seasons 2018/19 to 2021/22.

		Catalogue ID	Date (dd/mm/yy hh:mm:ss)	Average area scanned	SD	% of total image area
2018 to 2019	1	A0100103F00EAD00	07/12/18 13:41:23	133.02	0.78	74.06
	2	A010010401E42B00	09/01/19 14:05:49	96.60	0.33	90.45
	3	A01001042D99AF00	21/02/19 13:51:40	132.62	1.28	80.35
	4	A01001043EC12500	11/03/19 13:37:52	143.87	1.49	80.08
	5	A0100104547E2400	30/03/19 13:40:00	144.76	3.25	80.57
			average	130.17	19.63	80.27
2019 to 2020	7	A0100105575CF900	08/12/19 13:59:01	142.57	3.58	79.35
	8	A0100106B997E300	13/01/20 13:40:47	145.76	11.75	81.18
	9	A0100106B997E000	20/01/20 13:41:20	143.00	4.45	79.94
	10	A0100105D12FD000	24/03/20 13:53:00	141.78	2.76	79.92
	11	A0100105D1301600	24/03/20 13:52:46	5.52	0.18	65.96
			average	143.28	1.73	98.97
2021 to 2022	12	A0300106C0789B00	15/11/21 13:40:17	112.25	17.71	63.59
	13	A0300106C0E62900	22/11/21 13:50:17	109.38	24.47	60.95
	14	A0300106C35BA300	30/12/21 13:50:12	108.85	9.45	60.57
	15	A0300106C3E65F00	11/01/22 13:39:53	62.02	1.82	81.88
	16	A0300106C3E65000	16/01/22 13:19:40	76.97	20.95	62.19
	17	A0300106C5118800	04/02/22 13:18:00	122.25	29.17	68.04
	18	A0300106C9817600	16/03/22 13:45:57	142.27	1.79	79.72
			average	104.86	27.10	67.13
			multi season average	115.50	37.92	74.64

Average areas scanned (km^2^) calculated from each observer’s scanned areas, which varied due to individuals’ determination of the bay’s edge alongside the proportion of the scanned area to the total footprint of the image.

### Detection and labelling of whales

Images were pansharpened using the ESRI algorithm, and the resulting 4-band images were analysed in ESRI ArcMap v10.6 for images from 2018 to 2020, and in ArcPro v2.8.0 for the 2021 to 2022 season (www.esri.com/). Entire images were systematically scanned by multiple observers using a 0.0125 km^2^ grid and identified features of interest (FOIs) recorded as ESRI point shapefiles, with the point being placed on the centre of the FOI^3^. Observer contribution is detailed in Table 2. Due to logistical limitations, different observers were involved in different seasons. For consistency, a single observer was involved in the scanning of all three seasons. As whales do not always present themselves clearly at the surface, with their whole body clearly visible from overhead, identification is often based on singular or multiple presented cues.

**Table 2:** Individual observer (i to viii) contribution over the three seasons of very high resolution image analysis of Wilhelmina Bay.

Shaded ‘$\times$’ indicates that the observer analysed the image, whilst an unshaded ‘−’ indicates that the observer was not involved in the analyses of the indicated image.

Identified FOIs were first reviewed prior to full classification, with obvious non-whale FOIs removed. Candidate features were initially assigned to either ‘yes’ or ‘no’ whale category based on an instantaneous classification. This is akin to the decision process that occurs during traditional line-transect ship-borne surveys, where observers rely on their judgement and undefined cues to determine the validity of a sighting. Only those initially classified as likely (‘yes’) to be a whale were then classified fully.

**Fig. 3:**
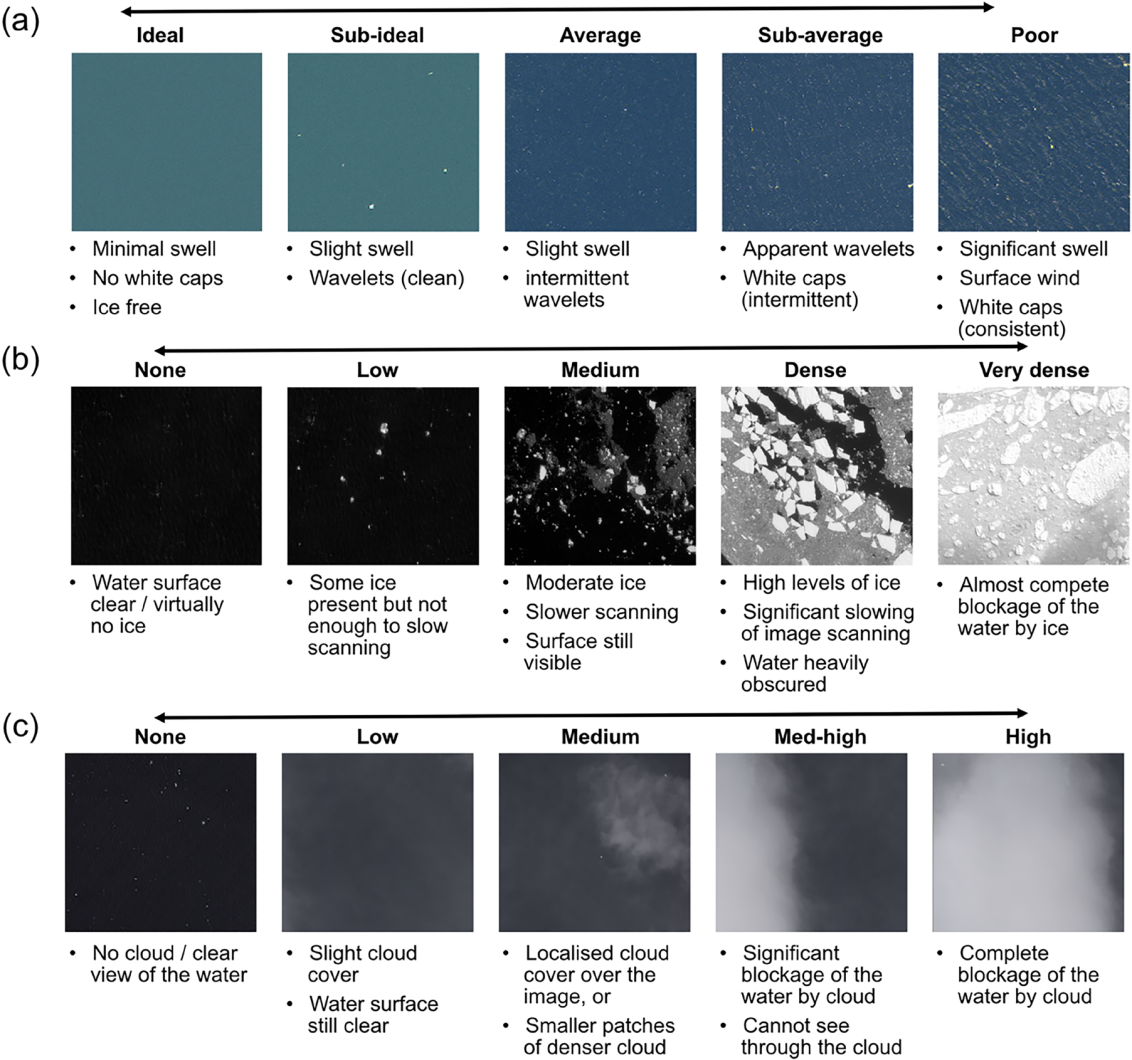
Localised environmental condition scales recorded at the site of each FOI for (**a**) sea state; (**b**) sea ice levels; and (**c**) cloud cover. Satellite imagery © Maxar Technologies.

Full classification involved scoring the FOIs based on the criteria set out in Cubaynes, *et al*.^3^ and equation and weighting factors from Bamford, *et al*.^4^, whereby FOIs scoring >9 were classified as ‘Definite’ whales; >7 as ‘probable’ whales and <7 as ‘unclassified’. For clarity, unclassified FOIs represent features that whilst detected by the observers, and therefore scored in some criteria, do not reach a threshold where confidence in the identification of a whale can be assumed. Environmental conditions in the immediate vicinity of a classified FOI were recorded for sea state, sea ice levels and cloud cover following the scales set out in Fig. 3.

## Data Records

The presented data are available from the NERC UK Polar Data Centre^30^. This dataset contains two csv files: The first file, named ‘Identified_features.csv’, is the main dataset and details each individual FOI identified in the imagery by the analysts over the three seasons. These locations are independent of one another and represent the contribution of each analyst without any inter-analyst comparison. For ease of loading, the locations of the FOIs are provided by latitude and longitude positions in decimal degrees (ESPG: 4326) and attributed to the corresponding image and each observer. However, during the image annotation process multiple projection systems were used. The satellite imagery was provided by the supplier in a native WGS 1984 projection in UTM Zone 20S; no reprojection was conducted to avoid unnecessary processing and interpolation errors. However, to facilitate accurate spatial statistics, observers created their FOI shapefiles using a custom Lambert Azimuthal Equal Area (LAEA) projection with a central meridian of −62.25 and a latitude of origin of −64.649, centred on Wilhelmina Bay. These shapefiles were aligned to the satellite imagery, without reprojection, utilising on-the-fly projection. The LAEA projection was chosen as it provides more accurate areas and distances for spatial statistics on and within the vicinity of the FOIs. It is important the data frame coordinate system is set to the custom LAEA projection for the layers to load correctly over each other. The second file, named ‘Identified_features_description.csv’ provides information on the columns and naming conventions used in the main dataset. Within the main dataset we identified and present 819 candidate FOIs that represent baleen-whales or baleen-whale cues as observed in WV3 imagery across all seasons and all observers (Table 3). While the majority of these FOIs are likely humpback whales, definitive species ID in a mixed species environment is not currently possible^25^. For this reason, we did not assign FOIs to a specific species level.

**Table 3:** Number of Features of Interest (FOIs) identified in each season and image, pooled from all observers that passed the instantaneous ‘yes/no’ observer filter and were subsequently classified into the three levels of certainty.

	Image date (dd/mm/yyyy)	Definite	Probable	Unclassified	Totals	Season total
2018 to 2019	07/12/2018	0	7	58	65	
	09/01/2019	0	0	12	12	
	21/02/2019	22	10	43	75	
	11/03/2019	24	15	57	96	
	30/03/2019	5	10	25	40	288
2019 to 2020	08/12/2019	0	1	0	1	
	13/01/2020	0	0	13	13	
	20/01/2020	9	6	10	25	
	24/03/2020	7	18	38	63	102
2021 to 2022	15/11/2021	0	0	42	42	
	22/11/2021	0	1	55	56	
	30/12/2021	0	0	20	20	
	11/01/2022	1	1	209	211	
	16/01/2022	3	1	9	13	
	04/02/2022	0	0	56	56	
	16/03/2022	0	1	30	31	429
	Totals	71	71	677		819

## Technical Validation

### Certainty of whale identification

The key concepts pertaining to the technical validation of using satellite imagery to detect baleen whales have previously been discussed by Cubaynes and Fretwell^24^, and we direct the reader to their manuscript for further details. The data presented here have been screened for typographic errors and is provided in a csv format for wide accessibility. When viewing WorldView-3 satellite imagery, pansharpening of the higher resolution multispectral image is recommended for greater clarity. Whilst multiple pansharpening algorithms exist^31^, for comparability to the presented FOIs, we suggest that the ESRI algorithm is implemented as this has been observed to cause less pixel-shift related issues than occur when applying alternative algorithms^24^. However, we note that this algorithm is not openly available, which may present challenges for some users. In such cases, other, open-source algorithms exist and may be employed, although care should be taken to consider the impact of potential differences in image alignment.

## Usage Notes

All satellite imagery referenced here (Table 1) are available to be licenced from Maxar Technologies (formerly DigitalGlobe); see here for more information and access https://xpress.maxar.com/. Pricing is dependent on situation and usage, although all images listed are now available as archival imagery and are thus available at substantially lower price points than the original order. However, it is still recommended that interested parties contact Maxar Technologies for further information on specifics. It is also recommended that the same product level and type are acquired to prevent pixel-shift in alternative products distorting the positioning of the FOI points. We also reiterate the need to use the custom LAEA projection, detailed above, to set the data frame coordinate system to avoid alignment issues. We hope that these data can be used to reduce the burdensome manual effort often required to process aerial imagery for training data for machine learning algorithms.

## Data Availability

All data^30^ presented here are freely available from the NERC UK Polar Data Centre (10.5285/ab19aaba-12d6-44a7-89a3-7b45af0343ed).

## Code availability

No custom code was implemented during the generation of this dataset.
